# Bystander CPR Technique and Outcomes for Cardiac Arrest With and Without Opioid Toxicity

**DOI:** 10.1001/jamanetworkopen.2025.16340

**Published:** 2025-06-17

**Authors:** Brian Grunau, May Lee, Jane A. Buxton, Valerie Mok, Jennie Helmer, Andrew W. Tu, Sean van Diepen, Frank Scheuermeyer, Michael Asamoah-Boaheng, Ian R. Drennan, Steven C. Brooks, Laurie J. Morrison, Katie N. Dainty, Jim Christenson

**Affiliations:** 1 British Columbia Resuscitation Research Collaborative, British Columbia, Canada; 2 Faculty of Medicine, University of British Columbia, Vancouver, British Columbia, Canada; 3 Department of Emergency Medicine, University of British Columbia and St Paul’s Hospital, Vancouver, British Columbia, Canada; 4 Centre for Advancing Health Outcomes, St Paul’s Hospital, Vancouver, British Columbia, Canada; 5 British Columbia Emergency Health Services, Vancouver, British Columbia, Canada; 6 School of Population & Public Health, University of British Columbia, Vancouver, British Columbia, Canada; 7 British Columbia Coroners Service, Burnaby, British Columbia, Canada; 8 Department of Critical Care Medicine, University of Alberta, Edmonton, Alberta, Canada; 9 Department of Emergency Services, Sunnybrook Health Sciences Centre, Toronto, Ontario, Canada; 10 Ornge Critical Care Transport, Mississauga, Ontario, Canada; 11 Institute of Health Policy, Management and Evaluation, Dalla Lana School of Public Health, University of Toronto, Toronto, Ontario, Canada; 12 Department of Emergency Medicine, Queen’s University, Kingston, Ontario, Canada; 13 Division of Emergency Medicine, Department of Medicine, University of Toronto, Toronto, Ontario, Canada; 14 North York General Hospital, Toronto, Ontario, Canada; 15 Division of Cardiology, Department of Medicine, University of Alberta, Edmonton, Alberta, Canada; 16 Department of Public Health Services, Queen’s University, Kingston, Ontario, Canada

## Abstract

**Importance:**

Previous studies support bystander provision of chest compression–only cardiopulmonary resuscitation (CC-CPR) for out-of-hospital cardiac arrest (OHCA). However, it is unknown whether OHCA secondary to opioid toxicity may benefit from chest compression plus ventilation CPR (CCV-CPR).

**Objective:**

To examine the association between bystander CPR technique and outcomes among both opioid-associated OHCA (OA-OHCA) and otherwise undifferentiated OHCA.

**Design, Setting, and Participants:**

This cohort study (performed from August 1, 2023, to December 31, 2024) analyzed cases of adult emergency medical services–treated OHCA that occurred from December 1, 2014, to March 31, 2020, as identified through the British Columbia Cardiac Arrest Registry.

**Exposures:**

Cases were classified as OA-OHCA based on positive postmortem toxicologic investigations, death certificates, or opioid-specific hospital-based diagnoses. All other cases were classified as undifferentiated OHCA.

**Main Outcomes and Measures:**

Favorable neurologic outcome at hospital discharge (cerebral performance category ≤2). A multivariable Utstein-adjusted logistic regression model of complete cases was used to assess the association between bystander CPR technique (CC-CPR [reference] vs both CCV-CPR and no CPR individually) with outcomes. An interaction term between the OA-OHCA and bystander CPR technique was used to estimate associations among OA-OHCA and undifferentiated OHCA cases separately.

**Results:**

The study included 10 923 OHCAs. After removing 24 cases only treated with ventilatory support, there were 1343 OA-OHCAs (median [IQR] patient age, 40 [31-50] years; 1015 [76%] male) and 9556 undifferentiated OHCAs (median [IQR] patient age, 70 [58-81] years; 6636 (69%) male). In the OA-OHCA group, bystander CCV-CPR was associated with an increased odds of a favorable neurologic outcome (adjusted odds ratio [AOR], 2.85; 95% CI, 1.21-6.75) when compared with CC-CPR. No association was detected with favorable neurologic outcome (AOR, 1.52; 95% CI, 0.82-2.82) when no CPR was compared with CC-CPR. Among undifferentiated OHCAs, no association was detected with a favorable neurologic outcome (AOR, 1.16; 95% CI, 0.80-1.67) when CCV-CPR was compared with CC-CPR. No CPR was associated with a decreased odds of a favorable neurologic outcome (AOR, 0.69; 95% CI, 0.55-0.87) when compared with CC-CPR. The interaction term was statistically significant (*P *for interaction = .04).

**Conclusions and Relevance:**

In this cohort study of OHCA, bystander CCV-CPR (compared with CC-CPR) was associated with improved outcomes in opioid-associated OHCA; however, this association was not observed among undifferentiated cardiac arrests. These results suggest that the optimal bystander CPR technique for OA-OHCA and undifferentiated OHCA may differ and that ventilations may improve outcomes in OA-OHCA resuscitation.

## Key Points

**Question:**

Among adult patients experiencing out-of-hospital cardiac arrest, with either opioid-associated cardiac arrest or otherwise undifferentiated cardiac arrest, is chest compression plus ventilation cardiopulmonary resuscitation (CPR) associated with improved outcomes compared with chest compression–only CPR?

**Findings:**

In this cohort study of 1357 opioid-associated and 9556 undifferentiated out-of-hospital cardiac arrest cases, chest compression plus ventilation CPR was associated with improved outcomes in opioid-associated cardiac arrests but not in undifferentiated cardiac arrests.

**Meaning:**

These results support the practice of bystander provision of ventilatory support with CPR for opioid-associated cardiac arrests.

## Teaser

This cohort study examines the association between the bystander cardiopulmonary resuscitation technique and favorable neurologic outcomes among individuals experiencing opioid-associated and undifferentiated out-of-hospital cardiac arrest.

## Introduction

Opioid toxicity is an international public health issue and has been identified as a public health emergency in both the US and Canada.^1,2,3^ In the US, drug toxicity is one of the leading causes of death, accounting for 107 941 deaths in 2022, most of which were opioid related.^4^ In Canada, there are more than 5000 deaths due to opioid toxicity annually.^5^ Opioid toxicity can result in apnea and hypoxia and, without emergency intervention, can progress to cardiac arrest.

A recent study^6^ from Washington reported that opioid toxicity was implicated in 8.2% of out-of-hospital cardiac arrest (OHCA) cases. Current international guidelines on cardiopulmonary resuscitation (CPR) recommend that untrained lay rescuers initiate chest compression–only CPR (CC-CPR) when treating OHCAs.^7^ However, given that respiratory arrest and hypoxia are the pathophysiologic mechanisms implicated in opioid toxicity leading to cardiac arrest, some have advocated that the optimal bystander CPR strategy should differ from other OHCAs and that outcomes would be improved if bystanders performed chest compression plus ventilation CPR (CCV-CPR). However, there is no evidence to support this physiologic rationale,^8^ and there may be a risk of fewer bystanders intervening if the recommended treatment is more complicated and poses a risk of bodily fluid exposure.^9^

No data are currently available to inform the optimal CPR technique for opioid-associated OHCA (OA-OHCA) or whether the optimal CPR technique differs between those with OA-OHCA and otherwise undifferentiated (ie, all cases other than those that are associated with opioids) OHCA. The aim of this study was to examine the association between the bystander CPR technique and favorable neurologic outcomes among OA-OHCA and undifferentiated OHCA cases.

## Methods

### Study Design and Setting

We included cases from the British Columbia (BC) Cardiac Arrest Registry,^10^ which prospectively enrolls consecutive nontraumatic emergency medical service (EMS)–assessed OHCAs in the province of BC, under a waiver of informed consent. The BC population during the study period was approximately 5 million individuals, divided among 5 geographic health regions, served by 91 acute care hospitals.^11,12^ The registry follows international Utstein guidelines for data collection^13^; trained registry data staff manually review 911 dispatch records, EMS clinical records, defibrillator files, and hospital-based records and systematically abstract data into a REDCap (Research Electronic Data Capture) database.^14^ EMS clinical records included a dedicated cardiac arrest module for data collection based on Utstein elements, including bystander CPR interventions.^13^ Registry staff (who were unaware of this study) used hospital records to ascertain survival and neurologic status (using cerebral performance category) at hospital discharge.^13^ See the eMethods in Supplement 1 for further details. Ethical approval was obtained from the University of British Columbia–affiliated Providence Health Care Research Ethics Board. The study followed the Strengthening the Reporting of Observational Studies in Epidemiology (STROBE) reporting guideline.^15^

EMS OHCA care in BC is standardized^16^ and provided by a coordinated response of the provincial BC Emergency Health Services (BCEHS) and municipal fire and rescue departments initiated by a 911 call to a provincial dispatch center. BCEHS units are trained in basic life support (BLS), with some units additionally trained in advanced life support (ALS).^7^ Fire and rescue personnel are trained in BLS, including the use of automated external defibrillators.^7^ For a suspected OHCA, a fire and rescue unit, a BLS-trained BCEHS unit, and an advanced life support–trained BCEHS unit (if available) are dispatched. Medical care in BC is publicly funded and managed by the provincial government and documented for each resident with a unique provincial health number.

### Selection of Participants

We included consecutive OHCA in the BC Cardiac Arrest Registry from December 1, 2014, to March 31, 2020. We excluded (1) those unable to be matched to provincial databases (applicable for cases with no identifiers [eg, unknown name] and those whose home address is outside BC) or those deemed at risk of an incorrect match (due to conflicting data); (2) cases for which the patient had a previous OHCA during the study period; (3) cases in which the patient was younger than 18 years; (4) EMS-untreated cases (EMS attends but elects not to attempt resuscitation); and (5) EMS-witnessed cases (eMethods in Supplement 1).

### Data Linkages

We linked BC Cardiac Arrest Registry cases to multiple provincial data sets (using the unique provincial health number, name, birth date, and death date if applicable), describing all health care use (including inpatient and outpatient medical contacts, procedures performed, and medications prescribed), vital statistics (detailing death dates and cause of death), and BC Coroners Service investigations (eMethods in Supplement 1). Information on race and ethnicity is not collected as part of the Cardiac Arrest Registry.

### OA-OHCA, Exposure of Interest, and Outcome Definitions

OA-OHCA has been defined by the American Heart Association as cardiac arrest caused by the use of opioids, with or without cointoxicants.^8^ For this study, we classified cases as OA-OHCA if (1) the BC Coroners Service investigation (including routine biochemical testing for opioids) determined opioids to be relevant to the cause of death, (2) the death certificate documented an opioid toxicity-related cause of death, or (3) an opioid toxicity-related diagnosis was documented for the hospital stay. If opioid toxicity was documented in one of the sources but an alternate diagnosis known to cause OHCA was also documented in one of the sources (eg, acute coronary syndrome or coronary artery disease, intracranial hemorrhage, pulmonary embolism, sepsis, hemorrhage, aortic dissection, asphyxiation, or drowning^17^), this alternate cause was prioritized, and the case was classified as undifferentiated (eMethods in Supplement 1). All cases not fulfilling the definition of OA-OHCA were classified as undifferentiated OHCA.

The exposure of interest was the CPR technique performed by the bystander, categorized as CCV-CPR, CC-CPR, or no CPR. We planned to include a ventilation-only technique as a category; however, this was excluded due to a low number of cases. The primary and secondary outcomes were favorable neurologic outcome (defined as cerebral performance categories 1-2) and survival, both measured at hospital discharge.^13^

### Statistical Analysis

We performed analyses using SAS software version 9.4 (SAS Institute Inc) and R software, version 4.0.5 (R Project for Statistical Computing) from August 1, 2023, to December 31, 2024. We reported continuous variables as medians (IQRs) and categorical variables as numbers (percentages). We reported patient characteristics, categorized first by OA-OHCA vs undifferentiated OHCA, and then by bystander CPR technique. Due to the low number of ventilation-only cases (n = 24), these cases are not shown in the patient characteristic summaries.

For the primary analysis, we fit a multivariable logistic regression model to assess the association of bystander CPR technique with the primary outcome. We used CC-CPR as the reference category, given that it is the currently recommended intervention for untrained bystanders.^7^ We excluded cases with missing data required for adjustment covariates and cases with ventilation-only CPR, given the small number. We included an interaction term for OA-OHCA classification and bystander CPR technique in the primary analysis to calculate odds ratios (ORs) separately among the OA-OHCA and undifferentiated OHCA groups and to identify whether the association between bystander CPR technique and outcomes differed between strata defined by OA-OHCA. We included the following adjustment covariates in the model: age (continuous variable using natural cubic spline with 4 knots), sex, bystander witnessed status, location type (classified as public [including street, public building, place of recreation, airport, casino, and other public location] vs semipublic [including non–acute health care facility, nursing home or residential institution, and industrial site] vs private [including house, apartment or condominium, and other private location]), time of day (4 periods of 6 hours each, starting from midnight), episode period (divided into 3 periods: December 2014 to December 2016, January 2017 to December 2018, and January 2019 to March 2020), and the interval between the dispatch call and the first EMS unit arrival (continuous variable, in minutes). We adjusted for period given that the specific drugs involved and/or medical management may have changed over time. We did not include initial shockable rhythm in the model given that the initial cardiac rhythm is identified after the exposure of interest and thus may be affected by bystander CPR technique. Previous data^18^ have shown that initial shockable rhythms are more commonly found among those with bystander CPR (in both unadjusted and adjusted analyses), possibly due to bystander CPR reducing the ischemic insult and thus slowing the transition from ventricular fibrillation to nonshockable rhythms. Outcome probabilities were estimated based on the adjusted model for each bystander CPR technique and 95% CIs based on 1000 bootstrap samples.^19^ We also fit an unadjusted model. As a secondary analysis, we repeated the same models for the secondary outcome. Analyses used 2-sided tests with a significance level of *P*< .05.

We performed a sensitivity analysis to address missing data. We used fully conditional specification methods to impute 200 data sets to address missing data and refit the models.^20^ Ventilation-only cases were included for the multiple imputation procedures but excluded from the final models (eMethods in Supplement 1). Multiple imputation is a valid method to minimize bias that can result from excluding cases with missing data, even if the proportion with missing values is substantial, provided that data are missing at random.^21,22,23^ We described the characteristics of complete cases and those missing data required for the primary model.

## Results

### Patient Characteristics

**Figure 1.**
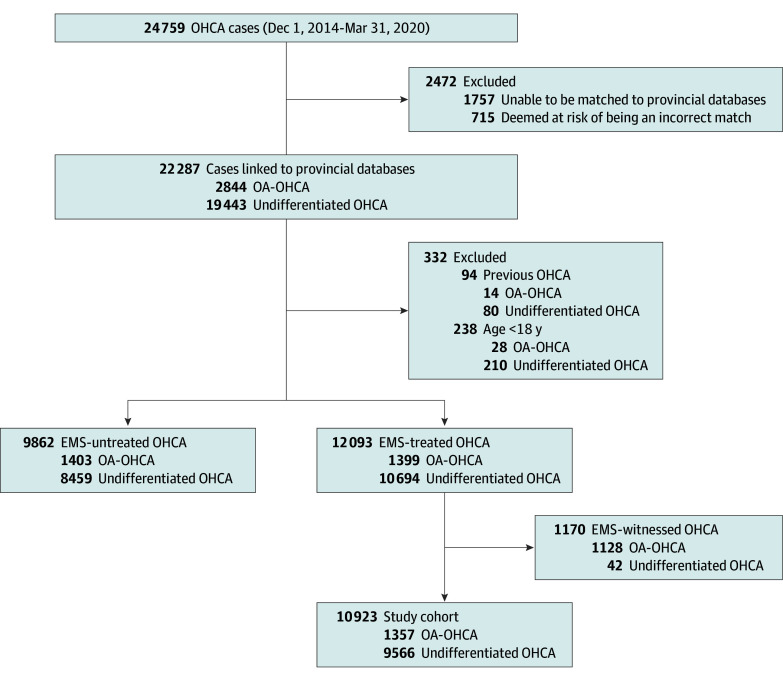
Study Cohort Flow EMS indicates emergency medical services; OA-OHCA, opioid-associated out-of-hospital cardiac arrest; OHCA, out-of-hospital cardiac arrest.

From December 1, 2014, to March 31, 2020, 24 759 cases were enrolled in the BC Cardiac Arrest Registry (Figure 1). The study included 10 923 OHCAs. After removing 24 cases only treated with ventilatory support, 10 899 OHCAs remained: 1343 OA-OHCAs (median [IQR] patient, 40 [31-50] years; 1015 [76%] male and 328 [24%] female; 1055 [79%] occurred in a private location) and 9556 undifferentiated OHCAs (median [IQR] patient age, 70 [58-81] years; 6636 [69%] male and 2911 [31%] female; 7530 [79%] occurred in a private location). Patient characteristics, categorized by OA-OHCA and undifferentiated OHCA, are given in eTable 1 in Supplement 1, and characteristics further categorized by bystander CPR technique are given in the Table. eTable 2 in Supplement 1 details the number of cases missing data according to bystander CPR technique.

**Table.** Characteristics of Study Cohort Categorized by OA-OHCA vs Undifferentiated OHCA and Bystander CPR Technique^a^^,^^b^

Characteristic	OA-OHCA				Undifferentiated OHCA			
	CCV-CPR (n = 82)	CC-CPR (n = 412)	Unknown (n = 295)^c^	No CPR (n = 554)	CCV-CPR (n = 355)	CC-CPR (n = 2496)	Unknown (n = 2828)^d^	No CPR (n = 3877)
Demographics								
Age, median (IQR), y	40 (31-51)	39 (30-50)	41 (31-49)	39 (31-51)	64 (54-76)	69 (58-81)	68 (57-79)	72 (60-82)
Sex								
Female	20 (24)	112 (27)	77 (26)	119 (22)	108 (30)	702 (28)	828 (29)	1273 (33)
Male	62 (76)	300 (73)	218 (74)	435 (79)	247 (70)	1793 (72)	1994 (71)	2602 (67)
Location of arrest								
Private	62 (77)	325 (79)	236 (80)	432 (78)	192 (54)	1927 (77)	2129 (76)	3282 (85)
Semiprivate	7 (9)	13 (3)	7 (2)	9 (2)	40 (11)	124 (5)	144 (5)	151 (4)
Public	12 (15)	74 (18)	52 (18)	110 (20)	122 (34)	442 (18)	542 (19)	436 (11)
EMS arrival interval, median (IQR), min^e^	6 (5-8)	6 (5-8)	6 (5-8)	6 (5-8)	7 (6-10)	7 (5-9)	7 (5-9)	7 (5-9)
Initial shockable cardiac rhythm	<6 (<7)	10 (2)	9 (3)	15 (3)	135 (38)	714 (29)	786 (28)	542 (14)
Bystander witnessed status								
Unwitnessed	61 (74)	346 (85)	236 (80)	468 (86)	138 (39)	1227 (49)	1411 (51)	2406 (63)
Bystander witnessed	21 (26)	61 (15)	58 (20)	77 (14)	216 (61)	1252 (51)	1380 (49)	1415 (37)
Bystander type								
Layperson	72 (92)	368 (95)	239 (96)	NA	289 (84)	2358 (96)	2308 (89)	NA
Police	<6 (<7)	15 (4)	7 (3)	NA	11 (3)	35 (1)	67 (3)	NA
Health care professional	<6 (<7)	6 (2)	<6 (<2)	NA	32 (9)	52 (2)	160 (6)	NA
Other	<6 (<7)	15 (4)	7 (3)	NA	11 (3)	35 (1)	67 (3)	NA
Time of day								
Midnight to 6 am	20 (24)	78 (19)	68 (23)	114 (21)	36 (10)	309 (12)	338 (12)	546 (14)
6 am to noon	15 (18)	101 (25)	75 (26)	133 (24)	104 (29)	745 (30)	859 (30)	1286 (33)
Noon to 6 pm	23 (28)	119 (29)	73 (25)	149 (27)	121 (34)	809 (32)	893 (32)	1133 (29)
6 pm to midnight	15 (18)	101 (25)	75 (26)	133 (24)	104 (29)	745 (30)	859 (30)	1286 (33)
Cardiac and pulmonary diagnosis before OHCA^f^								
Hypertension	6 (7)	50 (12)	32 (11)	56 (10)	149 (43)	1253 (51)	1352 (49)	2049 (53)
Diabetes	7 (9)	30 (7)	11 (4)	41 (8)	89 (26)	716 (29)	823 (30)	1240 (32)
Prior MI	0 (0)	<6 (<1)	7 (2)	6 (1)	30 (9)	213 (9)	245 (9)	334 (9)
Prior PCI	0 (0)	<6 (<1)	<6 (<2)	0 (0)	12 (3)	92 (4)	112 (4)	140 (4)
Prior CABG	<6 (<7)	<6 (<1)	0 (0)	<6 (<1)	<6 (<2)	35 (1)	39 (1)	49 (1)
Prior stroke	0 (0)	6 (1)	<6 (<2)	<6 (<1)	13 (4)	95 (4)	123 (4)	198 (5)
Atrial fibrillation	<6 (<7)	8 (2)	<6 (<2)	<6 (<1)	20 (6)	259 (11)	282 (10)	459 (12)
Chronic heart failure	<6 (<7)	16 (4)	9 (3)	15 (3)	57 (17)	573 (23)	630 (23)	1041 (27)
Chronic kidney disease	13 (16)	42 (10)	29 (10)	59 (11)	61 (18)	570 (23)	591 (21)	1044 (27)
COPD	8 (10)	45 (11)	43 (15)	68 (13)	61 (18)	403 (16)	457 (17)	822 (21)
Cancer	<6 (<7)	18 (4)	10 (3)	27 (5)	54 (16)	430 (18)	475 (17)	785 (20)
Mental health diagnosis before OHCA^f^								
Mood and anxiety disorders	38 (47)	226 (56)	152 (53)	283 (52)	84 (24)	549 (22)	621 (22)	904 (24)
Depression	29 (36)	217 (54)	145 (51)	257 (47)	83 (24)	520 (21)	550 (20)	821 (21)
Schizophrenia or delusional disorders	12 (15)	63 (16)	29 (10)	90 (17)	17 (5)	85 (3)	113 (4)	154 (4)
Substance use disorder	39 (48)	209 (52)	152 (53)	276 (51)	38 (11)	248 (10)	287 (10)	470 (12)
Cardiac medications before OHCA^g^								
β-Blocker	<6 (<7)	24 (6)	18 (6)	36 (7)	92 (27)	771 (31)	853 (31)	1343 (35)
ACEI or ARB	<6 (<7)	39 (10)	26 (9)	43 (8)	123 (36)	1050 (43)	1144 (41)	1707 (44)
Lipid-modifying agent	<6 (<7)	27 (7)	15 (5)	27 (5)	109 (32)	895 (37)	1018 (37)	1530 (40)
Antithrombotic	<6 (<7)	15 (4)	10 (3)	21 (4)	89 (26)	716 (29)	800 (29)	1284 (33)
Warfarin	0 (0)	<6 (<1)	<6 (<2)	6 (1)	26 (8)	199 (8)	202 (7)	375 (10)
Antiarrhythmic drugs	0 (0)	<6 (<1)	<6 (<2)	<6 (<1)	<6 (<2)	59 (2)	62 (2)	103 (3)
Psychiatric medications before OHCA^g^								
Antidepressants	28 (35)	182 (45)	116 (40)	238 (44)	98 (28)	578 (24)	690 (25)	1042 (27)
Antipsychotics	23 (28)	137 (34)	74 (26)	170 (31)	48 (14)	319 (13)	350 (13)	556 (14)
Anxiolytics or benzodiazepines	13 (16)	84 (21)	58 (20)	114 (21)	61 (18)	392 (16)	383 (14)	643 (17)
Opioid medications before OHCA^g^								
Opioids	29 (36)	207 (51)	130 (45)	263 (48)	80 (23)	657 (27)	734 (27)	1191 (31)
Non-OAT	25 (31)	168 (41)	84 (29)	195 (36)	76 (22)	635 (26)	711 (26)	1160 (30)
Codeine	17 (21)	97 (24)	52 (18)	120 (22)	44 (13)	376 (15)	421 (15)	674 (18)
Fentanyl	<6 (<7)	<6 (<1)	0 (0)	<6 (<1)	<6 (<2)	17 (1)	18 (1)	29 (1)
Buprenorphine	<6 (<7)	26 (6)	<6 (<2)	34 (6)	<6 (<2)	11 (<1)	7 (<1)	17 (<1)
Hydromorphone	<6 (<7)	31 (8)	16 (6)	23 (4)	23 (7)	176 (7)	196 (7)	338 (9)
Morphine	<6 (<7)	20 (5)	11 (4)	18 (3)	7 (2)	57 (2)	67 (2)	123 (3)
Oxycodone	<6 (<7)	32 (8)	10 (3)	31 (6)	12 (3)	57 (2)	62 (2)	108 (3)
Tramadol	<6 (<7)	25 (6)	16 (6)	30 (6)	17 (5)	107 (4)	147 (5)	233 (6)
Other	0 (0)	<6 (<1)	<6 (<2)	<6 (<1)	0 (0)	<6 (<1)	<6 (<1)	6 (<1)
OAT	15 (19)	99 (24)	64 (22)	129 (24)	<6 (<2)	45 (2)	46 (2)	67 (2)
Methadone	11 (14)	71 (18)	47 (16)	89 (16)	<6 (<2)	40 (2)	33 (1)	44 (1)
Buprenorphine	7 (9)	51 (13)	29 (10)	65 (12)	<6 (<2)	8 (<1)	18 (1)	26 (1)
Extended-release morphine	<6 (<7)	<6 (<1)	7 (2)	7 (1)	0 (0)	<6 (<1)	9 (<1)	9 (<1)

Abbreviations: ACEI, angiotensin-converting enzyme inhibitor; ARB, angiotensin receptor blockers; CABG, coronary artery bypass graft; CC-CPR, chest compression–only cardiopulmonary resuscitation; CCV-CPR, chest compression plus ventilation cardiopulmonary resuscitation; COPD, chronic obstructive pulmonary disease; CPR, cardiopulmonary resuscitation; EMS, emergency medical service; MI, myocardial infarction; NA, not applicable; OA-OHCA, opioid-associated out-of-hospital cardiac arrest; OAT, opioid agonist therapy; OHCA, out-of-hospital cardiac arrest; PCI, percutaneous intervention.

^a^ Data are presented as number (percentage) unless otherwise indicated. Those receiving ventilation-only bystander CPR are not shown here due to the low case counts (14 OA-OHCA and 10 undifferentiated OHCA).

^b^ Numbers less than 6 are suppressed due to local privacy regulations to protect against individual reidentification.

^c^ Among the 295 OA-OHCA cases, 292 (99%) received bystander CPR with an unknown technique, and 3 cases were missing data on whether bystander CPR was attempted. Those receiving unknown bystander CPR technique were excluded from the primary and secondary analyses.

^d^ Among the 2828 undifferentiated cases, 2780 (98%) received bystander CPR with an unknown technique, and 48 were missing data on whether bystander CPR was attempted. Those receiving unknown bystander CPR technique were excluded from the primary and secondary analyses.

^e^ Measured from the time the 911 call was answered at dispatch.

^f^ Five years before date of cardiac arrest.

^g^ One year before date of cardiac arrest.

### Primary Analysis

**Figure 2.**
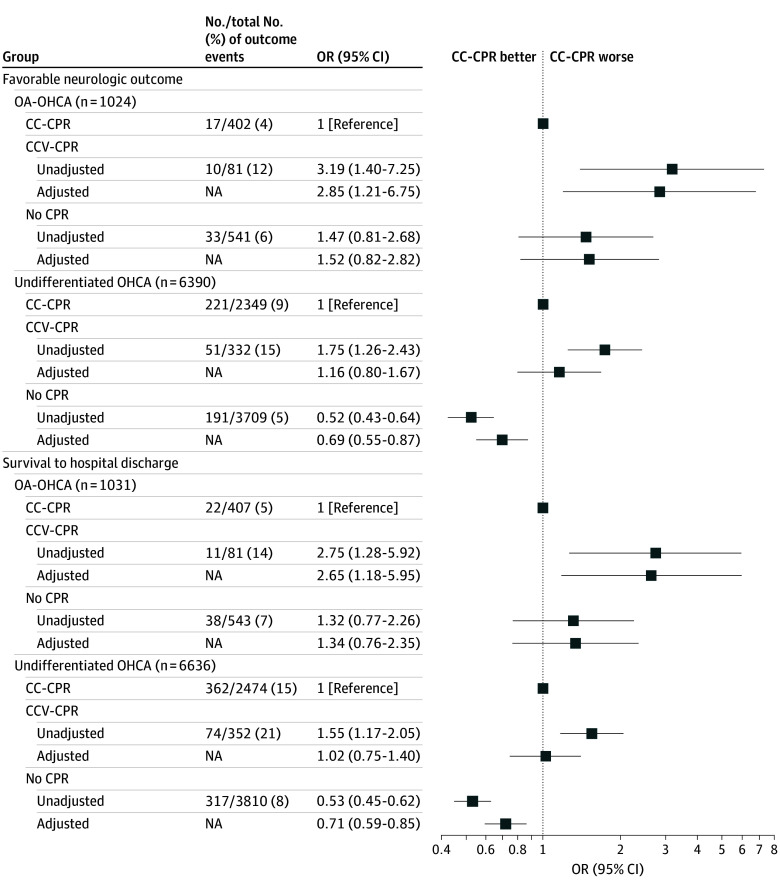
Association Between Bystander Cardiopulmonary Resuscitation (CPR) Technique and Favorable Neurologic Outcomes and Survival to Hospital Discharge Among OA-OHCA and Undifferentiated OHCA Cases (Complete Case Analysis) Model adjusted for age, sex, location of arrest, time of day, arrest year, bystander witnessed status, and interval between dispatch call and first emergency medical services unit arrival. Error bars indicate 95% CIs. CC-CPR indicates chest compression–only cardiopulmonary resuscitation; CCV-CPR, chest compression plus ventilation cardiopulmonary resuscitation; NA, not applicable; OA-OHCA, opioid-associated out-of-hospital cardiac arrest; OR, odds ratio.

Figure 2 shows unadjusted and adjusted results of the logistic regression models (complete case analysis, n = 7414) examining the outcome of favorable neurologic outcome at hospital discharge. The interaction term for OA-OHCA classification and bystander CPR technique was statistically significant (*P* for interaction = .04).

In the OA-OHCA group, bystander CCV-CPR was associated with an increased odds of a favorable neurologic outcome (adjusted OR [AOR], 2.85; 95% CI, 1.21-6.75) when compared with CC-CPR. No association was detected with favorable neurologic outcome (AOR, 1.52; 95% CI, 0.82-2.82) when no CPR was compared with CC-CPR. Among those treated with CC-CPR, the estimated adjusted probability of a favorable neurologic outcome was 4.4% (95% CI, 2.5%-6.6%) compared with 10.8% (95% CI, 5.1%-17.3%) among those treated with CCV-CPR (difference, 6.4 percentage points; 95% CI, 0.5-12.9 percentage points) and 6.3% (95% CI, 4.3-8.5%) among those treated with no CPR (difference, 2.0 percentage points; 95% CI, −1.0 to 4.8 percentage points).

Among undifferentiated OHCAs, no association was detected with a favorable neurologic outcome (AOR, 1.16; 95% CI, 0.80-1.67) when CCV-CPR was compared with CC-CPR. No CPR was associated with a decreased odds of a favorable neurologic outcome (AOR, 0.69; 95% CI, 0.55-0.87) when compared with CC-CPR. Among those treated with CC-CPR, the estimated adjusted probability of a favorable neurologic outcome was 9.4% (95% CI, 8.2%-10.6%) compared with 10.5% (95% CI, 8.0%-13.1%) among those treated with CCV-CPR (difference, 1.1 percentage points; 95% CI, −1.6 to 3.8 percentage points) and 7.0% (95% CI, 6.1%-7.9%) among those treated with no CPR (difference, −2.0 percentage points; 95% CI, −3.8 to −0.9 percentage points).

### Secondary Analysis

Results of models examining the outcome of survival to hospital discharge were consistent with the primary analysis (Figure 2). The interaction term for OA-OHCA classification and bystander CPR technique was statistically significant (*P* for interaction = .04).

### Sensitivity Analyses

**Figure 3.**
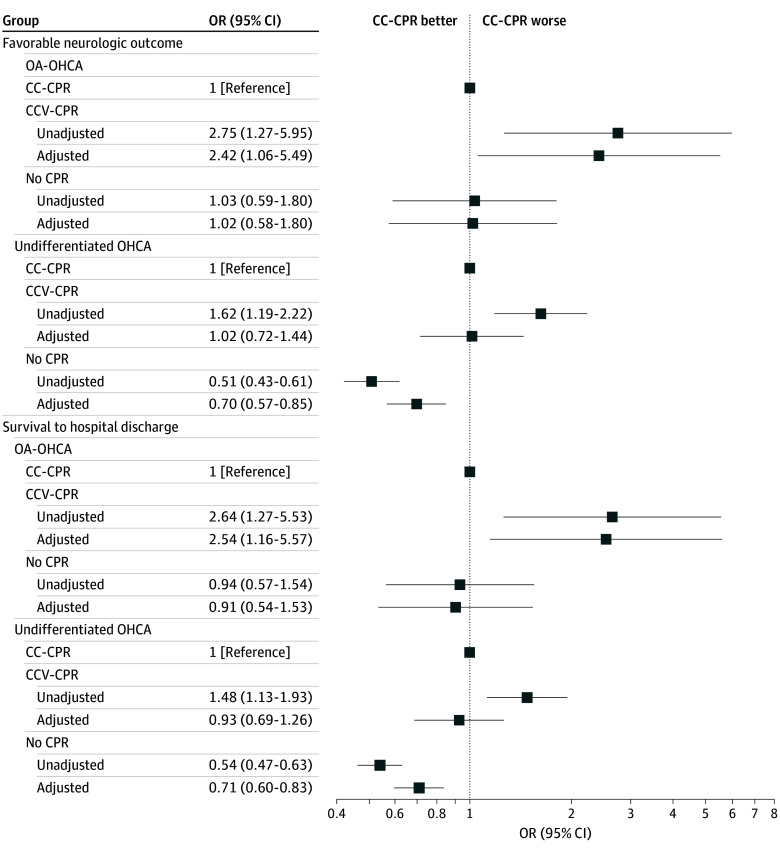
Multiple Imputation Models to Assess the Association Between Bystander Cardiopulmonary Resuscitation (CPR) Technique and Favorable Neurologic Outcomes at Hospital Discharge and Survival to Hospital Discharge Among OA-OHCA and Undifferentiated OHCA Cases Models adjusted for age, sex, location of arrest, time of day, arrest year, bystander witnessed status, and interval between dispatch call and first emergency medical services unit arrival. Error bars indicate 95% CIs. CC-CPR indicates chest compression–only cardiopulmonary resuscitation; CCV-CPR, chest compression plus ventilation cardiopulmonary resuscitation; OA-OHCA, opioid-associated out-of-hospital cardiac arrest; OR, odds ratio.

Results of models using multiple imputation (n = 10 923) are shown in Figure 3, which demonstrated results similar to the complete cases models. eTable 3 in Supplement 1 lists the characteristics of complete cases and cases with missing data for the primary analysis that were addressed with multiple imputation.

## Discussion

This prospective, observational cohort study included more than 10 000 OHCA cases with linkages to multiple provincial data sources to identify cases due to opioid toxicity. Among those with OA-OHCA, compared with bystander-performed CC-CPR, CCV-CPR was associated with improved favorable neurologic outcomes, but no bystander CPR was not significantly associated with worse outcomes. Conversely, among undifferentiated OHCAs, compared with CC-CPR, CCV-CPR was not associated with improved outcomes, but no bystander CPR was associated with worse outcomes. Overall, these data indicate that the association between bystander CPR technique and patient outcomes differs between those with OA-OHCA and other OHCAs and that ventilatory support for OA-OHCAs may be important for bystander CPR to be impactful.

There is a paucity of existing evidence to inform management of opioid-associated cardiac arrest. Previous data on bystander CPR techniques have examined undifferentiated OHCAs, most of which are due to primary cardiac causes.^17^ While OHCA of a primary cardiac origin results from a sudden cessation of cardiac output (when the body is fully oxygenated), the pathophysiologic mechanism of OA-OHCA is fundamentally different, resulting from respiratory depression, hypoxia, and secondary cardiac arrest.^8^ Whereas in an OHCA with a primarily cardiac cause compressions can move oxygenated blood to vital organs, in OA-OHCA interventions to oxygenate deoxygenated blood may be particularly critical. In this study, bystander CCV-CPR for OA-OHCA was associated with improved outcomes compared with CC-CPR. Interestingly, CC-CPR did not appear to be superior to no CPR.

Among undifferentiated OHCAs, we did not detect an association between CCV-CPR (compared with CC-CPR) and improved outcomes. This finding is consistent with previous literature suggesting that ventilatory support provided by bystanders does not appear to provide additional benefit compared with chest compressions alone.^24^ For adult OHCA, current guidelines recommend that untrained rescuers perform chest compression–only CPR,^7^ which became the recommended approach in 2010.^25^ A 2000 trial from Washington randomized 520 patients to dispatch-instructed CC-CPR or CCV-CPR and reported that survival to hospital discharge was 14.6% and 10.4%, respectively (*P* = .18).^26^ A 2010 clinical trial from Sweden randomized 1276 witnessed OHCAs (after excluding intoxication cases) to dispatcher-instructed CC-CPR or CCV-CPR and reported 30-day survival in 8.7%, and 7.0%, respectively (*P* = .29).^24^ A subsequent similar trial performed by Rea et al^27^ in Washington and London reported survival to hospital discharge in 12.5% and 11.0%, respectively (*P* = .31). A meta-analysis of these trials concluded that dispatch-instructed CC-CPR increased the chance of survival by 22% (number needed to treat of 41).^28^ In the trial by Rea et al,^27^ bystanders randomized to CC-CPR appeared to comply more frequently compared with CCV-CPR (80.5% vs 72.7%), suggesting that part of the benefit in CC-CPR may be that bystanders are simply more willing to perform this intervention.^9,29^ An observational study in Japan of more than 40 000 witnessed OHCAs reported that CCV-CPR, compared with CC-CPR, was associated with improved 30-day favorable neurologic outcomes, especially for younger patients with noncardiac causes and those with CPR delays,^30^ suggesting that some phenotypes may benefit from CCV-CPR.

A recent American Heart Association scientific statement^8^ described OA-OHCA as having distinct pathophysiologic and epidemiologic mechanisms compared with other OHCA, thus requiring targeted guidelines, but with substantial knowledge gaps to inform optimal management.^8^ An obstacle highlighted for research was accurately identifying cases of OA-OHCA. International registry data collection guidelines recommend that drug toxicity–related cases be classified generally as “drug overdose,” thus including an array of unintentional and intentional drug poisonings of prescribed and unprescribed substances.^13,31^ Historically, drug-related cases were thought to make up a small proportion of OHCA; however, recent data suggest that drug toxicity may be responsible for up to 10% of OHCAs in North America.^6,32^ In this study, OHCA case data were linked to multiple provincial data sets to systematically classify OA-OHCA cases. However, applying these data to patient care in real time may be difficult given that on-scene information is often lacking to ascertain the OHCA origin. Further study is required to develop methods to identify OA-OHCA cases using data available in the prehospital setting.

Although the aim of this study was not to compare OA-OHCA with otherwise undifferentiated cases, it offers an opportunity to examine the substantial differences between these groups. Compared with undifferentiated cases, the OA-OHCA group more often had unwitnessed cases, had fewer cases with initial shockable rhythms, and had few previous cardiopulmonary diagnoses but had more cases with mental health diagnoses. With a median age of only 40 years and the high incidence of this condition (in 2023, 81 083 and 8480 opioid-related deaths were documented in the US^33^ and Canada,^5^ respectively), the prospect of improving outcomes for OA-OHCA highlights the potential for years of life that can be gained. This study provides data to inform the optimal bystander treatment of OA-OHCA and may inform CPR training programs. However, further study is still needed to enhance our knowledge of how to best manage these critically ill patients.

### Limitations

This study has limitations. It was observational, thus causality cannot be inferred. Our results may not be generalizable to regions with differing patient characteristics, medical management, and/or nonprescription drug supply. Although standard Utstein variables were used for adjustment, there may have been additional unmeasured confounders. For example, those who provided CCV-CPR may have been more knowledgeable or skilled at CPR than those providing CC-CPR. Our data included cases during 2016 to 2020. Although it is possible that the association between bystander CPR techniques and outcomes may differ for more contemporary cases, this is unlikely.

Our classification of OA-OHCA may not have been correct for all cases. Bystander CPR technique was recorded by EMS professionals; there may have been errors in this determination, and bystander technique may not have been consistent throughout the bystander resuscitation effort. There was a substantial proportion of missing data for bystander CPR technique. Among those with missing bystander CPR technique data, more than 98% were known to have had bystander CPR performed but were missing the technique used. These cases demonstrated characteristics similar to other cases, suggesting that there were few systematic differences from those with nonmissing data. We implemented the multiple imputation method to address missing data, and results were consistent with the main analysis. The group of undifferentiated OHCA cases included all cases not classified as opioid related; some of these cases may benefit from CCV-CPR (eg, those with drowning, hanging, or other respiratory etiologies); however, this was not investigated in this study.

## Conclusions

Among patients experiencing OA-OHCA, CCV-CPR was associated with a higher odds of favorable neurologic outcomes at hospital discharge compared with CC-CPR. This association was not detected among patients experiencing otherwise undifferentiated OHCA. This study suggests that the optimal bystander CPR technique for OA-OHCA and undifferentiated OHCA may differ and supports the practice of bystander provision of ventilatory support during CPR for opioid-associated cardiac arrests.

## Notes

Supplement 1.
eMethods.
**eTable 1. **Missing Values for Characteristics of cases, categorized by OA-OHCA vs. Undifferentiated OHCA, and grouped by CPR technique **eTable 2. **Characteristics of Study Cohort, Categorized by OA-OHCA vs. Undifferentiated OHCA **eTable 3. **Characteristics of Study Cohort, Categorized by OHCA with Complete Case vs. with Cases with Missing Data
eReferences

Supplement 2.
Data Statement
