# Supplementary material for: Bystander CPR Technique and Outcomes for Cardiac Arrest With and Without Opioid Toxicity
Source: JAMA Netw Open. 2025 Jun 17;8(6):e2516340. doi: 10.1001/jamanetworkopen.2025.16340 (PMC12175020; doi:10.1001/jamanetworkopen.2025.16340)
Supplement: Supplement 1. — eMethods. eTable 1. Missing Values for Characteristics of cases, categorized by OA-OHCA vs. Undifferentiated OHCA, and grouped by CPR technique eTable 2. Characteristics of Study Cohort, Categorized by OA-OHCA vs. Undifferentiated OHCA eTable 3. Characteristics of Study Cohort, Categorized by OHCA with Complete Case vs. with Cases with Missing Data eReferences [file jamanetwopen-e2516340-s001.pdf]

Supplemental Online Content

Grunau B, Lee M, Buxton JA, et al. Bystander CPR technique and outcomes for cardiac arrest with and without opioid toxicity. *JAMA Netw Open.* 2025;8(6):e2516340. doi:10.1001/jamanetworkopen.2025.16340

eMethods.

eTable 1. Missing Values for Characteristics of cases, categorized by OA-OHCA vs. Undifferentiated OHCA, and grouped by CPR technique

eTable 2. Characteristics of Study Cohort, Categorized by OA-OHCA vs. Undifferentiated OHCA

eTable 3. Characteristics of Study Cohort, Categorized by OHCA with Complete Case vs. with Cases with Missing Data

eReferences

This supplemental material has been provided by the authors to give readers additional information about their work.

© 2025 Grunau B et al. *JAMA Network Open*.

eMethods

a. The BC Cardiac Arrest Registry Methodology

The British Columbia (BC) Cardiac Arrest Registry prospectively enrolls emergency medical system (EMS)-assessed non-traumatic out-of-hospital cardiac arrest (OHCA), and has been operating since December 2005. "EMS" is defined as professionals dispatched by 9-1-1 operators to an OHCA, in BC which includes provincial BC Emergency Health Services units and municipal fire-rescue department units. A OHCA is defined as cases located in the out-of-hospital environment for which: (1) EMS personnel perform chest compressions; (2) an AED applied by a bystander or EMS delivered a shock; or, (3) the patient is determined to be pulseless but no treatments are provided. Cases are classified as "treated" if EMS provided treatments of any kind. Other than "trauma" (defined as penetrating, blunt, or burn related injuries), all other OHCAs are enrolled in the registry, including those with no obvious cause, anaphylaxis, drowning, drug or chemical poisoning, electrocution, extremes of cold or heat, foreign body obstruction, hanging or mechanical suffocation/strangulation, non-traumatic exsanguination, venomous stings/bites, and smoke inhalation.

The registry prospectively identifies all cases that could possibly be a cardiac arrest, through a sensitive process including: (1) alerts from 9-1-1 dispatch based on dispatch codes; (2) a review of all files generated from use of defibrillators; (3) daily and monthly surveillance reports of electronical medical records including vital signs, diagnostic codes, bystander interventions documented, disposition (including transported to hospital, discharged from scene, or died), and a mandatory question within the EMS clinical record (asked for all patient encounters) "did the patient experience a cardiac arrest at any time before or during contact?". Registry staff manually review approximately four times the number of cases that are eventually deemed a OHCA and are included in the registry.

Trained registry staff collect data according to international guidelines, using the Utstein template,[1] including: provincial health number, age, sex, street address and OHCA location type (classified as public [including street, public building, place of recreation, airport, casino, other public location], semi-public [including non-acute healthcare facility, nursing home/residential institution, industrial site], vs. private [including house, apartment/condo, other private location]), witnessed status (by bystander, EMS, or none), bystander CPR technique (chest compression-plus-ventilation cardiopulmonary resuscitation [CPR], chest compression-only CPR, CPR performed but technique type unknown, ventilation-only CPR, no CPR), bystander AED application +/- defibrillation, and time-stamped EMS events including scene arrival, interventions performed (including defibrillations and airway management), and medications administered. Registry staff use hospital records to classify outcomes of survival and neurological status using cerebral performance category (CPC) at hospital discharge.[2]

The registry commenced in December 2005 and initially covered approximately three quarters of BC's population.[3] In 2019 the registry footprint was expanded to include the entire province. The registry includes approximately 350 data variables per case, with data collected during a manual review of records from: 9-1-1 dispatch, fire-rescue clinical records, BCEHS clinical records, prehospital defibrillators, and hospital sources. Historically, EMS clinical records were paper-based and included a cardiac arrest section (based on Utstein elements[1]) which specifically included data fields detailing whether bystanders performed: ventilations, chest compressions, and/or AED application. During 2019-2020 BCEHS transitioned to an electronical medical record, which continued to include a mandatory cardiac arrest module based on Utstein elements, however a question regarding whether bystanders specifically performed ventilations within CPR was no longer included as a required data field.

b. Data Linkage Procedures to Provincial Datasets

The BC Cardiac Arrest Registry includes personal identifiers (including name, date of birth, provincial health number, and date of death [if applicable]) that were used for linkage to external databases for this study, facilitated by Population Data BC. Population Data BC is a multi-university, data and education resource, governed by the BC Government Ministry of Health, that coordinates and provides data linkages for research projects in BC. Population Data BC uses a combination of deterministic and probabilistic linkages, and in cases of competing matches, clerical review to link data from different sources.[4] The software used was developed by Population Data BC following the data linkage theories of Newcombe[5] and operationalized by Fellegi and Sunter,[6] and Jaro and Winkler.[7,8]

© 2025 Grunau B et al. *JAMA Network Open*.

Access to data provided by the Data Stewards is subject to approval but can be requested for research projects through the Data Stewards or their designated service providers. The following data sets were used in this study: the BC Cardiac Arrest Registry, BC Coroners Service, the Consolidation File (demographics, registry), Hospital Discharge Abstract Database (DAD), Medical Services Plan (MSP), PharmaNet, Vital Statistics, and the National Ambulatory Care Reporting System (NACRS). Further information regarding this project and these data sets is available by visiting the Population Data BC project webpage at: https://my.popdata.bc.ca/project_listings/21-049/collection_approval_dates. All inferences, opinions, and conclusions drawn in this publication are those of the author(s), and do not reflect the opinions or policies of the Data Steward(s).

For this project, Population Data BC facilitated linkage of OHCAs in the BC Cardiac Arrest Registry[9] to the following provincial datasets:

i. **Consolidation File** lists basic demographic, geographic and registration information for residents in BC for BC's public health insurance plan. We utilize registration dates to identify when patients in the analytic cohort are residents in BC.

ii. **Discharge Abstract Database (DAD)** details all hospitalizations, including admission and discharge dates, most responsible diagnosis and secondary diagnoses (including noting whether the diagnoses were present prior to hospital admission or occurred while in hospital), procedures, and vital status at discharge. This source provided data on pre-cardiac arrest patient comorbidities which were identified using primary or secondary diagnoses associated with any healthcare encounter in the DAD (within five years preceding the index cardiac arrest event), using validated International Classification of Diseases code definitions.

iii. **Medical Services Plan (MSP)** database tracks all BC physicians' service billing and includes records of up to three diagnostic codes per physician-patient encounter. This source provided pre-cardiac arrest comorbidity information, as well as hospital-based diagnoses. We included data from MSP within five years preceding the index cardiac arrest event.

iv. **PharmaNet** tracks details of dispensed medications from privately- or publically-operated community or hospital outpatient pharmacies (prescriber, dispenser, facility, and drug). We included data from Pharmanet within 1 year preceding the index cardiac arrest event.

v. **BC Vital Statistics** registers all deaths in BC, that occur in- or out-of-hospital. Vital statistics records include cause of death, which is obtained from the Medical Certificate of Cause of Death, which is completed by a physician, nurse practitioner, or the coroner.

vi. **National Ambulatory Care Reporting System (NACRS)** collects ambulatory care data for emergency departments (EDs), day surgery, and medical and surgical day clinics within hospitals, including patient demographic and clinical data (e.g., presenting complaint and discharge diagnosis). Low volume emergency departments in BC do not contribute data to this database.

vii. The **BC Coroner Service**[10] investigates all unnatural, sudden and unexpected deaths in BC, including deaths related to unregulated drug toxicity. For this study, the BC Coroners Service only provided data for deaths secondary to non-prescription drug use. Investigations include data from the following sources, as appropriate: interviews with next of kin and individuals at the scene of death, medical record evaluations, examination of the body, autopsy, and blood and urine testing. All deaths suspected to be possibly due to drug toxicity undergo testing for a panel of substances (including opioids, stimulants, benzodiazepines, psychedelics, prescription and over-the-counter drugs, etc.). All cases for which the BC Coroners Service deemed the cause of death due to non-prescription drug toxicity were linked to the study dataset, including data of the implicated substance(s).

c. Classification of Cases as "Opioid-Associated Out-of-Hospital Cardiac Arrest"

Step 1: Each case was evaluated with the following criteria to determine if a case satisfied the definition of OA-OHCA:

© 2025 Grunau B et al. *JAMA Network Open*.

i. **BC Coroners Service**: If any of the following drugs were identified in drug testing and determined (by the Coroners Service through case investigation) to be relevant to the cause of death, the case was classified as an OA-OHCA: 3-Methylfentanyl, 4-Fluoroisobutyryl Fentanyl, 6-Monoacetylmorphine, Acetyl Fentanyl, Buprenorphine, Carfentanil, Codeine, Cyclopropyl Fentanyl, EDDP, Fentanyl, Furanylfentanyl, Hydrocodone, Hydromorphone, Methadone, Morphine, Norfentanyl, Oxycodone, U-47700, W-18. The BC Coroners Service only provided data of deaths secondary to non-prescription drugs.

ii. **BC Vital Statistics - Death**: A case was identified as OA-OHCA, if any of the following ICD-10-CA codes were used to identify the cause of death: T40.1-T40.4, T40.6, Y45.^, F11.^. These capture all opioid-related deaths (non-prescription and prescription toxicity).

iii. **Hospital-Based Diagnostic codes**: Hospital-based diagnostic codes (including DAD, NACRS, and MSP codes) were used, according to previous standards: diagnostic codes indicative of a hospitalization due to opioid use have been previously established.[11] Cases were classified as an OA-OHCA if any of the following opioid-specific diagnostic codes were registered: ICD-9: 304.0, 304.7, 305.5, 965.0, 970.1; ICD-10-CA: T40.1-T40.4, T40.6, Y45.^, F11.^. These capture all opioid-related deaths (non-prescription and prescription toxicity).

Step 2: However, given that alternate causes of OHCA may actually be the primary etiology even if opioids are implicated in a case, we created a list of alternate diagnoses (and corresponding ICD-10 codes) *a priori* that would supersede an initial OA-OHCA classification. We created this list based on previous data which has described the etiologies of OHCA (e.g., acute coronary syndrome/coronary artery disease, intracranial hemorrhage, pulmonary embolism, sepsis, hemorrhage, aortic dissection, asphyxiation/drowning).[9] For example, if diagnostic codes for a cases were "opioid overdose" and "cardiac arrest", this would be classified as an OA-OHCA. However, if the diagnostic codes for a case included "opioid overdose", "cardiac arrest", and "aortic dissection", then the case would be classified as "undifferentiated" (as the aortic dissection diagnosis would be prioritized).

To identify alternate etiology diagnoses that would supersede an OA-OHCA classification, we examined: (1) pre-admission (i.e., pathology present prior to hospital arrival) hospital diagnosis codes (from NACRS and DAD); and, (2) Vital Statistics Cause of Death codes. Using this list of alternate diagnoses, the following ICD-10-CA codes were identified in cases initially classified as OA-OHCA (from *Step 1*), but were thereby re-classified as "undifferentiated": A04.^ (Other bacterial intestinal infections), A40.^ (Streptococcal sepsis), A41.^ (Other sepsis), A49.^ (Bacterial infection of unspecified site), B95.^ (Streptococcus and staphylococcus as the cause of diseases classified to other chapters), B96.^ (Other bacterial agents as the cause of diseases classified to other chapters), D62.^ (Acute posthemorrhagic anemia), I21.^ (Acute myocardial infarction), I24.^ (Other acute ischemic heart diseases), I25.^ (Chronic ischaemic heart disease), I26.^ (Pulmonary embolism), I31.^ (Other diseases of pericardium), I33.^ (Acute and subacute endocarditis), I38.^ (Endocarditis, valve unspecified), I45.^ (Other conduction disorders), I49.^ (Other cardiac arrhythmias), I60.^ (Subarachnoid hemorrhage), I62.^ (Other nontraumatic intracranial hemorrhage), I70.^ (Atherosclerosis), I71.^ (Aortic aneurysm and dissection), I72.^ (Other aneurysm and dissection), I73.^ (Other peripheral vascular diseases), I74.^ (Arterial embolism and thrombosis), I77.^ (Other disorders of arteries and arterioles), I79.^ (Disorders of arteries, arterioles and capillaries in diseases classified elsewhere), I82.^ (Other venous embolism and thrombosis), J13.^ (Pneumonia due to Streptococcus pneumoniae), J14.^ (Pneumonia due to Haemophilus influenzae), J15.^ (Bacterial pneumonia, not elsewhere classified), J18.^ (Pneumonia, organism unspecified), S02.^ (Fracture of skull and facial bones), S05.^ (Injury of eye and orbit), S06.^ (Intracranial injury), S12.^ (Fracture of neck), S15.^ (Injury of blood vessels at neck level), S22.^ (Fracture of rib(s), sternum and thoracic spine), S26.^ (Injury of heart), S27.^ (Injury of other and unspecified intrathoracic organs), T17.^ (Foreign body in respiratory tract), W65.^ (Drowning and submersion while in bath-tub), W66.^ (Drowning and submersion following fall into bath-tub), W69.^ (Drowning and submersion while in natural water), W74.^ (Unspecified drowning and submersion), X00.^ (Exposure to uncontrolled fire in building or structure).

Overall, of the 1357 cases finally classified as OA-OHCA for the analysis (after *Step 1* and *2*), 955 (70.4%), 239 (17.6%), and 163 (12.0%) cases were classified based on data from BC Coroners Service, BC Vital Statistics, and Hospital-Based Diagnostic Codes, respectively.

© 2025 Grunau B et al. *JAMA Network Open*.

d. Provincial Datasets Linkage summary

Among the 24,759 OHCA cases from December 1, 2014 to March 31, 2020, a total of 23,002 (93%) cases (those who had available identifiers and were residents of BC) were linked by Population Data BC. Inconsistencies between selected data fields with the BC Cardiac Arrest Registry and external datasets were examined to remove any cases that could not be confirmed to be a correct match. These included discrepancies between age with the Consolidation File, ED registration date in NACRS, hospital admission date in DAD, and vital status in Vital Statistics Death registry or Coroners Services. After excluding 715 cases due to these inconsistencies, overall a total of 22,287 / 24,759 (90%) BC Cardiac Arrest Registry OHCA cases remained.

e. Multiple Imputation Modeling Methods

SAS 9.4 PROC MI was used to generate 200 imputations with 25 burn-in iterations using the fully conditional specification method for the cohort, which included cases with bystander ventilation-only CPR (n=10,923). Separate imputation datasets were created for each outcome which also included the outcome of interest in the imputation models.

For the outcome, survival to hospital discharge, the imputation datasets were created in a 2-step process to maintain the correct data structure between whether a bystander attempted CPR, the type of bystander attempting CPR, and type of CPR that was attempted.

Step 1: The discriminant function method was used to impute the categorical variables: time of day (00:01 – 06:00, 06:01 – 12:00, 12:01-18:00, 18:01-24:00), sex (male, female), location of arrest (public, semi, private), witnessed status (Yes, No), shockable initial rhythm (Yes, No) and, bystander CPR attempted (Yes, No). The time (in minutes) between dispatch call and first unit on scene was imputed using the regression method. Additionally, the year of arrest (2014 – 2016, 2017 – 2018, 2019 – 2020), age at arrest (in years), and the outcome, survival to hospital discharge (Yes, No), were included in the imputation modeling although these variables did not have any missing data.

Step 2: In cases where the bystander attempted CPR, the bystander type (lay person, police, healthcare, other) and CPR type (CC-CPR, CCV-CPR, no CPR) were imputed using the discriminant function method. All cases (n=10,923), including the 24 ventilation-only CPR cases, were used in the imputation models. This allowed for ventilation-only CPR to be a potential level for cases with unknown CPR technique; however, these cases were excluded before fitting the MI analysis model as done in the complete case analysis. All variables from Step 1 were included in the imputation model. Imputations were done separately for opioid-related and undifferentiated arrests to allow for potential differences in the distributions of the CPR types.

For the outcome, favourable neurological outcome (CPC 1/2, CPC 3/4/death), Step 1 as given above for the outcome survival to hospital discharge, was followed. For Step 2, the same procedure as described above was followed with the addition of imputing favourable neurological outcomes using the discriminant function method.

The imputation datasets were analyzed by fitting a logistic regression model for each of the outcomes, favourable neurological outcomes, and survival to hospital discharge, with the same adjustment variables used in the complete case analysis. Due to the low case count of bystander ventilation-only CPR, these cases were excluded from the analysis prior to model fitting as done in the complete case analysis. PROC MIANALYZE was used to pool parameter estimates and their corresponding standard errors on the logit scale using Rubin's rules and back-transformed to estimate odds ratios and confidence intervals.

© 2025 Grunau B et al. *JAMA Network Open*.

Supplemental Results

a. eTable 1: Characteristics of Study Cohort, Categorized by OA-OHCA vs. Undifferentiated OHCA

	Full Cohort (n=10,899)		Opioid-Associated OHCA (n=1343)		Undifferentiated OHCA (n=9556)	
	n or median	Missing n (%)	n or median	Missing n (%)	n or median	Missing n (%)
**Demographics**						
Age (years), median (IQR)	67 (5, 79)	0 (0)	40 (31, 50)	0 (0)	70 (58, 81)	0 (0)
Female, n (%)	3239 (29.7)	9 (0.1)	328 (24.4)	0 (0)	2911 (30.5)	9 (0.1)
Male, n (%)	7651 (70.2)		1015 (75.6)		6636 (69.4)	
Location of arrest		29 (0.3)		<6[1] (<0.4)		25 (0.3)
Private, n (%)	8585 (79.0)		1055 (78.8)		7530 (79.0)	
Semi, n (%)	495 (4.6)		36 (2.7)		459 (4.8)	
Public, n (%)	1790 (16.5)		248 (18.5)		1542 (16.2)	
EMS arrival interval[2] (min), median (IQR)	7 (5, 9)	20 (0.2)	6 (5, 8)	<6 (<0.4)	7 (5, 9)	19 (0.2)
Initial Shockable Cardiac Rhythm, n (%)	2215 (20.4)	26 (0.2)	38 (2.8)	<6 (<0.4)	2177 (22.8)	22 (0.2)
Witnessed status		126 (1.2)		15 (1.1)		111 (1.2)
Unwitnessed, n (%)	6293 (58.4)		1111 (83.7)		5182 (54.9)	
Bystander witnessed, n (%)	4480 (41.6)		217 (16.3)		4263 (45.1)	
Bystander type		4806 (44.1)		626 (46.6)		4180 (43.7)
Lay person, n (%)	5634 (92.5)		679 (94.7)		4955 (92.2)	
Police, n (%)	137 (2.2)		24 (3.3)		113 (2.1)	
Healthcare, n (%)	256 (4.2)		12 (1.7)		244 (4.5)	
Other, n (%)	66 (1.1)		<6 (<0.4)		64 (1.2)	
Time of day		12 (0.1)		<6 (<0.4)		10 (0.1)
0:00 - 6:00, n (%)	1509 (13.9)		280 (20.9)		1229 (12.9)	
6:00 - 12:00, n (%)	3318 (30.5)		324 (24.2)		2994 (31.4)	
12:00 - 18:00, n (%)	3320 (30.5)		364 (27.1)		2956 (31.0)	
18:00 - 24:00, n (%)	2740 (25.2)		373 (27.8)		2367 (24.8)	
**Cardiac/Pulmonary Dx prior to OHCA[3]**						
Hypertension, n (%)	4947 (46.2)	186 (1.7)	144 (11.0)	30 (2.2)	4803 (51.1)	156 (1.6)
Diabetes, n (%)	2957 (27.6)	185 (1.7)	89 (6.8)	28 (2.1)	2868 (30.5)	157 (1.6)
Prior MI, n (%)	840 (7.8)	187 (1.7)	18 (1.4)	30 (2.2)	822 (8.7)	157 (1.6)
Prior PCI, n (%)	362 (3.4)	188 (1.7)	6 (0.5)	30 (2.2)	356 (3.8)	158 (1.7)
Prior CABG, n (%)	132 (1.2)	189 (1.7)	<6 (<0.4)	30 (2.2)	128 (1.4)	159 (1.7)
Prior stroke, n (%)	445 (4.2)	189 (1.7)	16 (1.2)	30 (2.2)	429 (4.6)	159 (1.7)
Atrial fibrillation, n (%)	1036 (9.7)	189 (1.7)	16 (1.2)	30 (2.2)	1020 (10.9)	159 (1.7)
Chronic heart failure, n (%)	2343 (21.9)	189 (1.7)	42 (3.2)	30 (2.2)	2301 (24.5)	159 (1.7)
Chronic kidney disease, n (%)	2409 (22.5)	187 (1.7)	143 (10.9)	29 (2.2)	2266 (24.1)	158 (1.7)
Chronic obstructive pulmonary disease, n (%)	1907 (17.8)	185 (1.7)	164 (12.5)	28 (2.1)	1743 (18.5)	157 (1.6)
Cancer, n (%)	1801 (16.8)	187 (1.7)	57 (4.3)	29 (2.2)	1744 (18.6)	158 (1.7)
**Mental health diagnoses prior to OHCA[3]**						
Mood and anxiety disorders, n (%)	2857 (26.7)	184 (1.7)	699 (53.1)	27 (2)	2158 (23.0)	157 (1.6)
Depression, n (%)	2622 (24.5)	184 (1.7)	648 (49.2)	27 (2)	1974 (21.0)	157 (1.6)
Schizophrenia & delusional disorders, n (%)	563 (5.3)	189 (1.7)	194 (14.8)	30 (2.2)	369 (3.9)	159 (1.7)
Substance use disorder, n (%)	1719 (16.0)	185 (1.7)	676 (51.4)	28 (2.1)	1043 (11.1)	157 (1.6)
**Cardiac medications prior to OHCA[4]**						
Beta-blocker, n (%)	3140 (29.3)	171 (1.6)	81 (6.2)	26 (1.9)	3059 (32.5)	145 (1.5)
ACEi/ARB, n (%)	4137 (38.5)	167 (1.5)	113 (8.6)	26 (1.9)	4024 (42.7)	141 (1.5)
Lipid Modifying Agents, n (%)	3625 (33.8)	171 (1.6)	73 (5.5)	25 (1.9)	3552 (37.7)	146 (1.5)
Anti-thrombotics , n (%)	2940 (27.4)	172 (1.6)	51 (3.9)	26 (1.9)	2889 (30.7)	146 (1.5)
Warfarin, n (%)	811 (7.6)	175 (1.6)	9 (0.7)	26 (1.9)	802 (8.5)	149 (1.6)
Anti-arrhythmic drugs, n (%)	232 (2.2)	175 (1.6)	<6 (<0.4)	26 (1.9)	228 (2.4)	149 (1.6)
**Psychiatric medications prior to OHCA[4]**						
Antidepressants, n (%)	2972 (27.7)	166 (1.5)	564 (42.7)	23 (1.7)	2408 (25.6)	143 (1.5)

© 2025 Grunau B et al. *JAMA Network Open*.

Antipsychotics, n (%)	1677 (15.6)	172 (1.6)	404 (30.6)	24 (1.8)	1273 (13.5)	148 (1.5)
Anxiolytics / Benzodiazepines, n (%)	1748 (16.3)	171 (1.6)	269 (20.4)	25 (1.9)	1479 (15.7)	146 (1.5)
**Opioid medications prior to OHCA[4]**						
Opioids, n (%)	3291 (30.7)	169 (1.6)	629 (47.7)	24 (1.8)	2662 (28.3)	145 (1.5)
non-OAT, n (%)	3054 (28.5)	169 (1.6)	472 (35.8)	24 (1.8)	2582 (27.4)	145 (1.5)
Codeine, n (%)	1801 (16.8)	172 (1.6)	286 (21.7)	25 (1.9)	1515 (16.1)	147 (1.5)
Fentanyl, n (%)	73 (0.7)	176 (1.6)	7 (0.5)	26 (1.9)	66 (0.7)	150 (1.6)
Buprenophine, n (%)	103 (1.0)	176 (1.6)	66 (5.0)	26 (1.9)	37 (0.4)	150 (1.6)
Hydromorphone, n (%)	808 (7.5)	172 (1.6)	75 (5.7)	24 (1.8)	733 (7.8)	148 (1.5)
Morphine, n (%)	308 (2.9)	174 (1.6)	54 (4.1)	26 (1.9)	254 (2.7)	148 (1.5)
Oxycodone, n (%)	316 (2.9)	174 (1.6)	77 (5.8)	25 (1.9)	239 (2.5)	149 (1.6)
Tramadol, n (%)	576 (5.4)	176 (1.6)	72 (5.5)	26 (1.9)	504 (5.4)	150 (1.6)
Other, n (%)	16 (0.1)	176 (1.6)	<6 (<0.4)	26 (1.9)	12 (0.1)	150 (1.6)
OAT, n (%)	469 (4.4)	176 (1.6)	307 (23.3)	26 (1.9)	162 (1.7)	150 (1.6)
Methadone, n (%)	339 (3.2)	176 (1.6)	218 (16.6)	26 (1.9)	121 (1.3)	150 (1.6)
Buprenophine, n (%)	205 (1.9)	176 (1.6)	152 (11.5)	26 (1.9)	53 (0.6)	150 (1.6)
Kadian, n (%)	38 (0.4)	176 (1.6)	19 (1.4)	26 (1.9)	19 (0.2)	150 (1.6)

[1] Counts < 6 are suppressed due to local privacy regulations to protect against individual re-identification.
[2] Measured from the time the 9-1-1 call was answered at dispatch
[3] Diagnoses identified within 5 years prior to the date of cardiac arrest.
[4] Medical prescriptions identified within 1 year prior to the date of cardiac arrest

OA, opioid-associated; OHCA, out of hospital cardiac arrest; n, number; IQR, interquartile range; min., minute; Dx, diagnosis; ACEi/ARB, ace inhibitor/angiotensin receptor blocker; OAT, opioid agonist therapy; MI , myocardial infarction; PCI, percutaneous intervention; CABG, coronary artery bypass graft

© 2025 Grunau B et al. *JAMA Network Open*.

b. **eTable 2: Number of Missing Values for Characteristics of cases, categorized by OA-OHCA vs. Undifferentiated OHCA, and Bystander CPR technique**

	Opioid-Associated OHCA				Undifferentiated OHCA			
	CCV-CPR	CC-CPR	Unknown	No CPR	CCV-CPR	CC-CPR	Unknown	No CPR
**Demographics, n (%)**								
Age	0 (0)	0 (0)	0 (0)	0 (0)	0 (0)	0 (0)	0 (0)	0 (0)
Female Sex	0 (0)	0 (0)	0 (0)	0 (0)	<6 (<1)	6	<6 (<1)	0 (0)
Male Sex	0 (0)	0 (0)	0 (0)	0 (0)	0 (0)	<6 (<1)	6 (1)	<6 (<1)
Location of arrest	0 (0)	0 (0)	<6 (<1)	<6 (<2)	<6 (<1)	13 (<1)	8 (<1)	0 (0)
911 call to EMS arrival interval[1]	0 (0)	0 (0)	<6 (<1)	<6 (<2)	<6 (<1)	6 (<1)	10 (<1)	0 (0)
Initial Shockable Cardiac Rhythm	<6 (<2)	<6 (<2)	<6 (<1)	0 (0)	<6 (<1)	7 (<1)	12 (<1)	<6 (<2)
Witnessed status	<6 (<2)	<6 (<2)	9 (2)	<6 (<2)	17 (1)	37 (1)	56 (1)	<6 (<2)
Bystander type	23 (6)	45 (15)	N/A	11 (3)	45 (2)	247 (9)	N/A	23 (6)
Time of day	0 (0)	<6 (<2)	<6 (<1)	0 (0)	<6 (<1)	<6 (<1)	7 (<1)	0 (0)
**Cardiac/Pulmonary Dx prior to OHCA, n (%)[2]**								
Hypertension	8 (2)	9 (3)	12 (2)	10 (3)	46 (2)	65 (2)	35 (1)	8 (2)
Diabetes	7 (2)	9 (3)	11 (2)	10 (3)	48 (2)	65 (2)	34 (1)	7 (2)
Prior MI	8 (2)	9 (3)	12 (2)	10 (3)	48 (2)	64 (2)	35 (1)	8 (2)
Prior PCI	8 (2)	9 (3)	12 (2)	10 (3)	48 (2)	65 (2)	35 (1)	8 (2)
Prior CABG	8 (2)	9 (3)	12 (2)	10 (3)	48 (2)	66 (2)	35 (1)	8 (2)
Prior stroke	8 (2)	9 (3)	12 (2)	10 (3)	48 (2)	66 (2)	35 (1)	8 (2)
Atrial fibrillation	8 (2)	9 (3)	12 (2)	10 (3)	48 (2)	66 (2)	35 (1)	8 (2)
Chronic heart failure	8 (2)	9 (3)	12 (2)	10 (3)	48 (2)	66 (2)	35 (1)	8 (2)
Chronic kidney disease	8 (2)	9 (3)	11 (2)	10 (3)	47 (2)	66 (2)	35 (1)	8 (2)
Chronic obstructive pulmonary disease	8 (2)	8 (3)	11 (2)	10 (3)	47 (2)	65 (2)	35 (1)	8 (2)
Cancer	8 (2)	9 (3)	11 (2)	10 (3)	48 (2)	66 (2)	34 (1)	8 (2)
**Mental health Dx prior to OHCA, n (%)[2]**								
Mood and anxiety disorders	7 (2)	9 (3)	10 (2)	10 (3)	47 (2)	66 (2)	34 (1)	7 (2)
Depression	7 (2)	9 (3)	10 (2)	10 (3)	47 (2)	66 (2)	34 (1)	7 (2)
Schizophrenia & delusional disorders	8 (2)	9 (3)	12 (2)	10 (3)	48 (2)	66 (2)	35 (1)	8 (2)
Substance use disorder	7 (2)	9 (3)	11 (2)	10 (3)	47 (2)	65 (2)	35 (1)	7 (2)
**Cardiac medications prior to OHCA, n (%)[3]**								
Beta-blocker	7 (2)	8 (3)	10 (2)	10 (3)	44 (2)	60 (2)	31 (1)	7 (2)
ACEi/ARB	7 (2)	8 (3)	10 (2)	10 (3)	44 (2)	59 (2)	28 (1)	7 (2)
Lipid Modifying Agents	7 (2)	8 (3)	9 (2)	10 (3)	44 (2)	61 (2)	31 (1)	7 (2)
Anti-thrombotics	7 (2)	8 (3)	10 (2)	10 (3)	45 (2)	61 (2)	30 (1)	7 (2)
Warfarin	7 (2)	8 (3)	10 (2)	10 (3)	45 (2)	62 (2)	32 (1)	7 (2)
Anti-arrhythmic drugs	7 (2)	8 (3)	10 (2)	10 (3)	45 (2)	62 (2)	32 (1)	7 (2)
**Psychiatric medications prior to OHCA, n (%)[3]**								
Antidepressants	6 (1)	8 (3)	8 (1)	10 (3)	43 (2)	59 (2)	31 (1)	6 (1)
Antipsychotics	6 (1)	8 (3)	9 (2)	10 (3)	44 (2)	62 (2)	32 (1)	6 (1)
Anxiolytics / Benzodiazepines	6 (1)	8 (3)	10 (2)	9 (3)	44 (2)	61 (2)	32 (1)	6 (1)
**Opioid medications prior to OHCA, n (%)[3]**								
Opioids	6 (1)	8 (3)	9 (2)	9 (3)	45 (2)	60 (2)	31 (1)	6 (1)
non-OAT	6 (1)	8 (3)	9 (2)	9 (3)	45 (2)	60 (2)	31 (1)	6 (1)
Codeine	7 (2)	8 (3)	9 (2)	10 (3)	45 (2)	61 (2)	31 (1)	7 (2)
Fentanyl	7 (2)	8 (3)	10 (2)	10 (3)	45 (2)	62 (2)	33 (1)	7 (2)
Buprenophine	7 (2)	8 (3)	10 (2)	10 (3)	45 (2)	62 (2)	33 (1)	7 (2)
Hydromorphon	6 (1)	8 (3)	9 (2)	10 (3)	45 (2)	60 (2)	33 (1)	6 (1)
Morphine	7 (2)	8 (3)	10 (2)	9 (3)	45 (2)	61 (2)	33 (1)	7 (2)
Oxycodone	6 (1)	8 (3)	10 (2)	10 (3)	45 (2)	62 (2)	32 (1)	6 (1)
Tramadol	7 (2)	8 (3)	10 (2)	10 (3)	45 (2)	62 (2)	33 (1)	7 (2)
Other	7 (2)	8 (3)	10 (2)	10 (3)	45 (2)	62 (2)	33 (1)	7 (2)
OAT	7 (2)	8 (3)	10 (2)	10 (3)	45 (2)	62 (2)	33 (1)	7 (2)
Methadone	7 (2)	8 (3)	10 (2)	10 (3)	45 (2)	62 (2)	33 (1)	7 (2)
Buprenophine	7 (2)	8 (3)	10 (2)	10 (3)	45 (2)	62 (2)	33 (1)	7 (2)
Kadian	7 (2)	8 (3)	10 (2)	10 (3)	45 (2)	62 (2)	33 (1)	7 (2)

© 2025 Grunau B et al. *JAMA Network Open*.

OA, opioid-associated; OHCA, out of hospital cardiac arrest; CPR, cardiopulmonary resuscitation; n, number; CV-CPR, chest compression-plus-ventilation CPR; CC-CPR, chest compression-only CPR; Dx, diagnoses; CPR, cardiopulmonary resuscitation; ACEi/ARB, ace inhibitor/angiotensin receptor blocker; OAT, opioid agonist therapy; MI, myocardial infarction; PCI, percutaneous intervention; CABG, coronary artery bypass graft

© 2025 Grunau B et al. *JAMA Network Open*.

c. **eTable 3: Characteristics of Study Cohort, Categorized by OHCA with Complete Case vs. with Cases with Missing Data**

	Full Cohort[1] (n=10,899) n (%)	Complete Case[2] (n=7414) n (%)	Cases with Missing Data[3] (n=3485) n (%)	Number of Cases with Missing Data: Full Cohort n	Complete n	Cases with Missing n
**Demographics**						
Age (years), median (IQR)	67 (53, 79)	68 (53, 80)	66 (53, 77)	0	0	0
Female, n (%)	3239 (29.7)	2244 (30.3)	995 (28.6)	9	<6[4]	8
Male, n (%)	7651 (70.2)	5169 (69.7)	2482 (71.2)			
Location of arrest				29	0	29
Private, n (%)	8585 (79.0)	6019 (81.2)	2566 (74.2)			
Semi, n (%)	495 (4.6)	329 (4.4)	166 (4.8)			
Public, n (%)	1790 (16.5)	1066 (14.4)	724 (20.9)			
EMS arrival interval[5] (min), median (IQR)	7 (5, 9)	7 (5, 9)	7 (5, 9)	20	0	20
Initial Shockable Cardiac Rhythm, n (%)	2215 (20.4)	1243 (16.8)	972 (28.0)	26	11	15
Witnessed status				126	0	126
Unwitnessed, n (%)	6293 (58.4)	4565 (61.6)	1728 (51.4)			
Bystander witnessed, n (%)	4480 (41.6)	2849 (38.4)	1631 (48.6)			
Bystander type				4806	4329	477
Lay person, n (%)	5634 (92.5)	2920 (94.7)	2714 (90.2)			
Police, n (%)	137 (2.2)	62 (2)	75 (2.5)			
Healthcare, n (%)	256 (4.2)	87 (2.8)	169 (5.6)			
Other, n (%)	66 (1.1)	62 (2.0)	75 (2.5)			
Time of day				12	0	12
0:00 - 6:00, n (%)	1509 (13.9)	1066 (14.4)	443 (12.8)			
6:00 - 12:00, n (%)	3318 (30.5)	2299 (31)	1019 (29.3)			
12:00 - 18:00, n (%)	3320 (30.5)	2220 (29.9)	1100 (31.7)			
18:00 - 24:00, n (%)	2740 (25.2)	2299 (31.0)	1019 (29.3)			
**Cardiac/Pulmonary Dx prior to OHCA[6]**						
Hypertension, n (%)	4947 (46.2)	3422 (46.8)	1525 (44.9)	186	97	89
Diabetes, n (%)	2957 (27.6)	2052 (28.0)	905 (26.6)	185	96	89
Prior MI, n (%)	840 (7.8)	555 (7.6)	285 (8.4)	187	99	88
Prior PCI, n (%)	362 (3.4)	233 (3.2)	129 (3.8)	188	99	89
Prior CABG, n (%)	132 (1.2)	88 (1.2)	44 (1.3)	189	99	90
Prior stroke, n (%)	445 (4.2)	304 (4.2)	141 (4.2)	189	99	90
Atrial fibrillation, n (%)	1036 (9.7)	718 (9.8)	318 (9.4)	189	99	90
Chronic heart failure, n (%)	2343 (21.9)	1644 (22.5)	699 (20.6)	189	99	90
Chronic kidney disease, n (%)	2409 (22.5)	1740 (23.8)	669 (19.7)	187	97	90
Chronic obstructive pulmonary disease, n	1907 (17.8)	1353 (18.5)	554 (16.3)	185	97	88
Cancer, n (%)	1801 (16.8)	1262 (17.2)	539 (15.9)	187	97	90
**Mental health diagnoses prior to OHCA[7]**						
Mood and anxiety disorders, n (%)	2857 (26.7)	1996 (27.3)	861 (25.4)	184	94	90
Depression, n (%)	2622 (24.5)	1854 (25.3)	768 (22.6)	184	94	90
Schizophrenia & delusional disorders, n	563 (5.3)	408 (5.6)	155 (4.6)	189	99	90
Substance use disorder, n (%)	1719 (16.0)	1226 (16.8)	493 (14.5)	185	96	89
**Cardiac medications prior to OHCA[6]**						
Beta-blocker, n (%)	3140 (29.3)	2179 (29.8)	961 (28.2)	171	90	81
ACEi/ARB, n (%)	4137 (38.5)	2835 (38.7)	1302 (38.2)	167	86	81
Lipid Modifying Agents, n (%)	3625 (33.8)	2486 (33.9)	1139 (33.5)	171	89	82
Anti-thrombotics , n (%)	2940 (27.4)	2046 (27.9)	894 (26.3)	172	90	82
Warfarin, n (%)	811 (7.6)	580 (7.9)	231 (6.8)	175	92	83
Anti-arrhythmic drugs, n (%)	232 (2.2)	155 (2.1)	77 (2.3)	175	91	84
**Psychiatric medications prior to OHCA[6]**						
Antidepressants, n (%)	2972 (27.7)	2090 (28.5)	882 (25.9)	166	85	81
Antipsychotics, n (%)	1677 (15.6)	1206 (16.5)	471 (13.8)	172	88	84
Anxiolytics / Benzodiazepines, n (%)	1748 (16.3)	1245 (17.0)	503 (14.8)	171	89	82
**Opioid medications prior to OHCA[6]**						

© 2025 Grunau B et al. *JAMA Network Open*.

Opioids, n (%)	3291 (30.7)	2336 (31.9)	955 (28.1)	169	88	81
non-OAT, n (%)	3054 (28.5)	2178 (29.7)	876 (25.7)	169	88	81
Codeine, n (%)	1801 (16.8)	1278 (17.4)	523 (15.4)	172	90	82
Fentanyl, n (%)	73 (0.7)	54 (0.7)	19 (0.6)	176	92	84
Buprenophine, n (%)	103 (1.0)	91 (1.2)	12 (0.4)	176	92	84
Hydromorphone, n (%)	808 (7.5)	573 (7.8)	235 (6.9)	172	90	82
Morphine, n (%)	308 (2.9)	222 (3.0)	86 (2.5)	174	91	83
Oxycodone, n (%)	316 (2.9)	234 (3.2)	82 (2.4)	174	91	83
Tramadol, n (%)	576 (5.4)	395 (5.4)	181 (5.3)	176	92	84
Other, n (%)	16 (0.1)	12 (0.2)	<6 (<0.2)	176	92	84
OAT, n (%)	469 (4.4)	343 (4.7)	126 (3.7)	176	92	84
Methadone, n (%)	339 (3.2)	246 (3.4)	93 (2.7)	176	92	84
Buprenophine, n (%)	205 (1.9)	150 (2.0)	55 (1.6)	176	92	84
Kadian, n (%)	38 (0.4)	22 (0.3)	16 (0.5)	176	92	84

[1] Study cohort excluding bystander ventilation-only CPR (n=24).
[2] Study cohort with complete case information used in the logistic regression model for favourable neurological outcome.
[3] "Cases with Missing Data" include cases missing any variables required for the regression model
[4] Counts < 6 are suppressed due to local privacy regulations to protect against individual re-identification.
[5] Measured from the time the 9-1-1 call was answered at dispatch.
[6] Diagnoses identified within 5 years prior to the date of cardiac arrest.
[7] Medical prescriptions identified within 1 year prior to the date of cardiac arrest.

OHCA, out of hospital cardiac arrest; n, number; IQR, interquartile range; min., minute; Dx, diagnosis; ACEi/ARB, ace inhibitor/angiotensin receptor blocker; OAT, opioid agonist therapy; MI , myocardial infarction; PCI, percutaneous intervention; CABG, coronary artery bypass graft

© 2025 Grunau B et al. *JAMA Network Open*.

eREFERENCES

1. Perkins GD, Jacobs IG, Nadkarni VM, et al. Cardiac Arrest and Cardiopulmonary Resuscitation Outcome Reports: Update of the Utstein Resuscitation Registry Templates for Out-of-Hospital Cardiac Arrest. *Circulation*. 2015;132(13):1286-1300. doi:10.1161/CIR.0000000000000144.

2. Perkins GD, Jacobs IG, Nadkarni VM, et al. Cardiac Arrest and Cardiopulmonary Resuscitation Outcome Reports: Update of the Utstein Resuscitation Registry Templates for Out-of-Hospital Cardiac Arrest. *Circulation*. 2015;132(13):1286-1300. doi:10.1161/CIR.0000000000000144.

3. Grunau B, Kawano T, Dick W, et al. Trends in care processes and survival following prehospital resuscitation improvement initiatives for out-of-hospital cardiac arrest in British Columbia, 2006–2016. *Resuscitation*. 2018;125:118-125. 10.1016/j.resuscitation.2018.01.049

4. Ark TK, Kesselring S, Hills B, McGrail KM. Population Data BC: Supporting population data science in British Columbia. *Int J Popul Data Sci*. 2020;4(2):1133. doi:10.23889/ijpds.v5i1.1133.

5. Newcombe H. *Handbook of Record Linkage: Methods for Health and Statistical Studies, Administration, and Business*. New York, NY: Oxford University Press, Inc; 1988.

6. Fellegi IP, Sunter AB. A Theory for Record Linkage. *J Am Stat Assoc*. 1969;64(328):1183-1210. doi:10.1080/01621459.1969.10501049.

7. Winkler W. Overview of record linkage and current research directions. In: *Technical Report Statistical Research Report Series RRS2006/02*. Washington D.C, USA: US Bureau of the Census; 2006:RRS2006/02.

8. Winkler W. String comparator metrics and enhanced decision rule in the Fellegi-Sunter model of record linkage. In: *Proceedings of the Section on Survey Research*. Washington, DC; 1990:354-359.

9. BC Cardiac Arrest Registry data (2020). *BC Resuscitation Research Collaborative* [publisher]. Data Extract. BC Resuscitation Research Collaborative (Approved 2021).

10. BC Coroners Service data (2020). *BC Coroners Service* [publisher]. Data Extract. BC Coroners Service (Approved 2021).

11. Weiss AJ, Heslin KC, Stocks C. ICD-9-CM and ICD-10-CM diagnosis codes defining opioid use disorder. In: *Healthcare Cost and Utilization Project (HCUP) Statistical Briefs*. Rockville, MD: Agency for Healthcare Research and Quality (US); 2006. https://www.ncbi.nlm.nih.gov/books/NBK557173/table/sb256.tab7/. Accessed June 15, 2023.

© 2025 Grunau B et al. *JAMA Network Open*.
