# Supplementary material for: Bystander CPR Technique and Outcomes for Cardiac Arrest With and Without Opioid Toxicity
Source: JAMA Netw Open. 2025 Jun 17;8(6):e2516340. doi: 10.1001/jamanetworkopen.2025.16340 (PMC12175020; doi:10.1001/jamanetworkopen.2025.16340)
Supplement: Supplement 2. — Data Statement [file jamanetwopen-e2516340-s002.pdf]

Data Sharing Statement

Grunau. Bystander CPR Technique and Outcomes for Cardiac Arrest With and Without Opioid Toxicity. *JAMA Netw Open*. Published June 17, 2025.
doi:10.1001/jamanetworkopen.2025.16340

Data
Data available: No

Additional Information
Explanation for why data not available: This has not been permitted by the research ethics board.
